# Intermittent claudication caused by a Masson tumour of the thigh: case report

**DOI:** 10.1093/jscr/rjag524

**Published:** 2026-07-06

**Authors:** Yongkun Fang, Zhaolei Chen

**Affiliations:** Department of Vascular Surgery, Department of General Surgery, Northern Jiangsu People's Hospital Affiliated to Yangzhou University, 98 Nantong West Road, Yangzhou, China; Department of Vascular Surgery, Department of General Surgery, Northern Jiangsu People's Hospital Affiliated to Yangzhou University, 98 Nantong West Road, Yangzhou, China

**Keywords:** Masson’s tumour, intermittent claudication, superficial femoral artery

## Abstract

Masson’s tumor, also known as intravascular papillary endothelial hyperplasia, is a rare condition characterized by nonspecific clinical manifestations, which frequently lead to its misidentification as malignant neoplasms such as sarcoma. A definitive diagnosis is established through histopathological examination. This report describes a case of a patient presenting with a mass in the left thigh accompanied by intermittent claudication of the ipsilateral lower limb, who was subsequently diagnosed with Masson’s tumour​ following surgical intervention.

## Introduction

Masson’s tumour, also known as intravascular papillary endothelial hyperplasia (IPEH), is a relatively rare vascular lesion that can occur in any anatomical site, with the head, neck, and extremities being the most commonly affected regions [1]. The clinical manifestations of IPEH are nonspecific, often leading to confusion with other soft tissue space-occupying lesions. Diagnosis is typically established through histopathological examination. This article aims to enhance understanding of this condition by reporting a case of Masson’s tumour involving the superficial femoral artery and reviewing relevant literature.

## Case report

**Figure 1:**
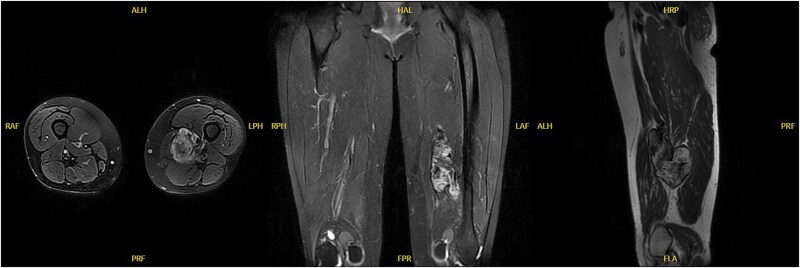
MRI revealed a lobulated mass.

**Figure 2:**
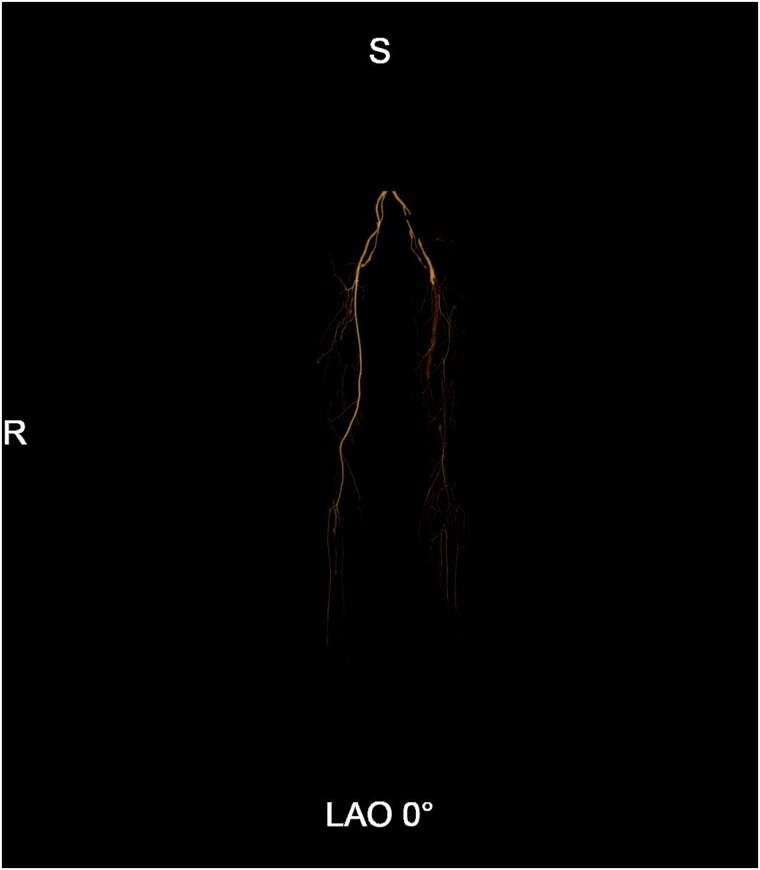
CT angiography revealed occlusion of the left femoral and popliteal arteries, with distal vessels visualized intermittently.

**Figure 3:**
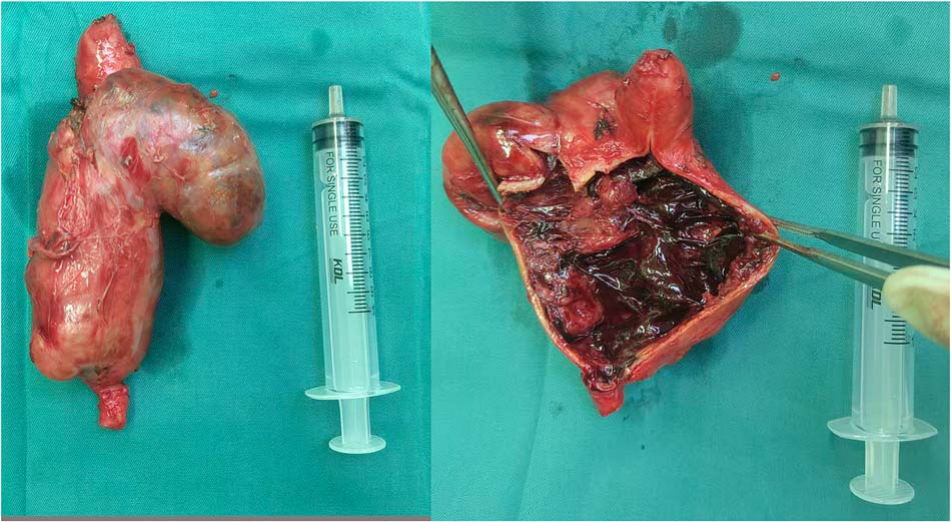
Intraoperatively resected mass and its cross-sectional view.

**Figure 4:**
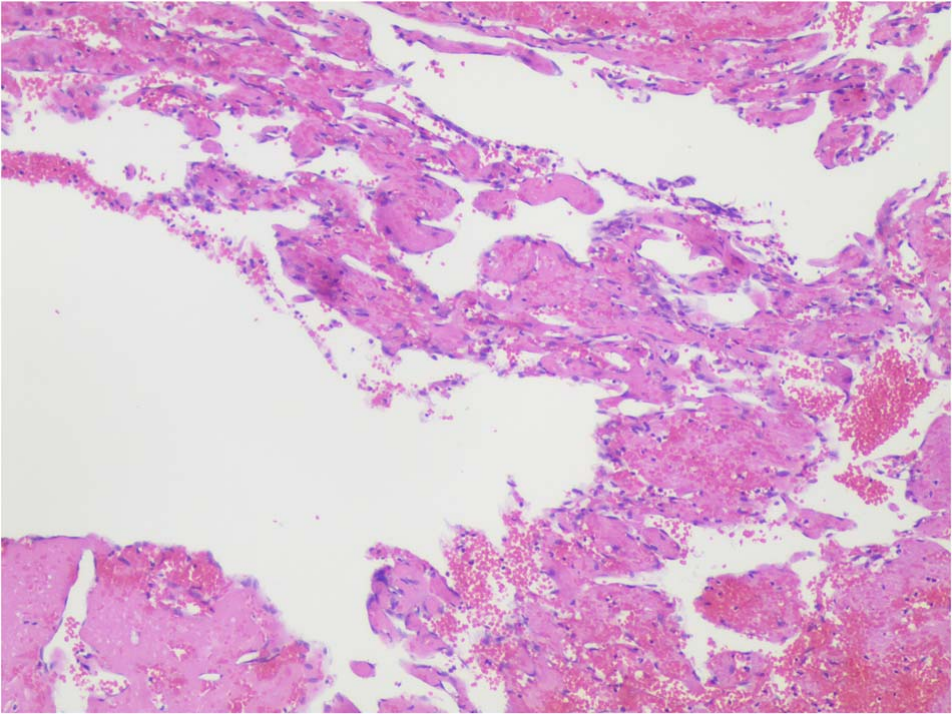
Vascular proliferation accompanied by organized thrombus, with focal papillary hyperplasia of endothelial cells (HE*400).

A middle-aged woman accidentally discovered a painless mass, approximately the size of an egg, in the medial aspect of her left thigh 17 years ago. She did not undergo any treatment, and the mass slowly increased in size over time. The patient reported a sensation of heaviness and easy fatigue in her left lower limb. One week prior to presentation, she experienced soreness, numbness, and pain in her left calf after walking ~100 meters, which improved with rest. She also noted decreased skin temperature in the left lower limb. Physical examination revealed a firm, palpable mass measuring ~8 × 5 cm in the medial aspect of the left thigh. The skin temperature of the left leg was lower, and the popliteal artery pulse was not palpable in the left lower extremity. Vascular ultrasound of both lower limbs indicated a heterogeneous echogenic mass in the left lower limb, which appeared to communicate with the superficial femoral artery, raising suspicion for Masson’s tumour. Magnetic resonance imaging (MRI) demonstrated a space-occupying lesion in the posteromedial muscle group of the left thigh, with possible hemorrhage. The course of the left deep femoral artery appeared thickened (Fig. 1). Computed tomography (CT) angiography revealed occlusion of the left femoral and popliteal arteries. The left anterior and posterior tibial arteries and the peroneal artery were visualized intermittently (Fig. 2). The patient underwent surgical resection. Intraoperative exploration revealed that the mass was connected to the superficial femoral artery. The segments of the superficial femoral artery proximal and distal to the mass were hardened, non-elastic, and lacked arterial pulsation. Following mass excision, a Fogarty embolectomy catheter exploration identified occlusion of the distal artery, which was difficult to recanalize. Consequently, the proximal and distal segments of the superficial femoral artery were ligated, and the surgery was concluded. The resected mass measured 90 × 66 × 51 mm. Upon sectioning, the lesion showed significant intraluminal hyperplasia with extensive hemorrhage and thrombus formation (Fig. 3). Histopathological examination revealed proliferating blood vessels of varying sizes, accompanied by extensive hemorrhage and thrombus formation, partial organization of the thrombus, and focal papillary proliferation of endothelial cells (Fig. 4). Based on the clinical and histopathological findings, a diagnosis of Masson’s tumour was established. The patient recovered well postoperatively. The indication for bypass grafting will be determined by the development of lower limb ischemia.

## Discussions

Masson’s tumour was first described in 1923 by Pierre Masson based on his study of patients with ulcerated hemorrhoids. He proposed that this lesion primarily involves endothelial cell proliferation, followed by thrombus formation [2]. The etiology and risk factors of Masson’s tumor remain unclear; however, trauma and pre-existing local vascular abnormalities appear to be associated with its development. It is hypothesized that locally produced growth factors may stimulate endothelial cell hyperproliferation, potentially leading to the formation of Masson’s tumour [3]. The tumor can occur at any stage of life and shows no clear gender predilection [4], though some reports suggest a higher incidence in females compared to males [5]. The most commonly affected sites are the head, neck, and extremities. With increasing recognition of this entity, cases in other anatomical locations—such as the eyes, urethra, and kidneys—have also been documented [6–8]. Clinical manifestations are nonspecific and highly variable, ranging from asymptomatic presentations to localized masses, pain, or compressive symptoms, depending on the tumor site. Differentiating Masson’s tumor from other soft tissue masses can be challenging, and clinical diagnosis is often difficult. Imaging modalities such as ultrasound, CT, and MRI may aid in diagnosis [9], but definitive confirmation relies on histopathological evaluation. Histologically, Masson’s tumour is characterized by intravascular papillary structures associated with thrombus formation. These papillae consist of a connective tissue core lined by endothelial cells, without features of malignancy—such as cytologic atypia, mitotic activity, or necrosis [10]. Masson’s tumour is classified into three types: (i) Primary (pure) type: Arises within dilated vessels, most commonly veins. (ii) Secondary (mixed) type: Develops secondary to preexisting vascular lesions, such as varices, hemangiomas, pyogenic granulomas, or lymphangiomas. (iii) Extra-vascular (rare) type: Originates from organizing hematomas, which is exceedingly uncommon [5]. Surgical excision is generally curative, with a low recurrence rate and favorable prognosis, making it the preferred treatment approach.

## Conclusions

Masson’s tumour is a benign vascular lesion, yet its clinical presentation can be deceptive. When it occurs deep in the lower extremities and compresses a major artery, it can lead to severe intermittent claudication and is highly susceptible to misdiagnosis as a malignant tumor before surgery. Enhancing awareness of this condition, along with early recognition and complete surgical resection, is crucial for accurate diagnosis and curative treatment.

## References

[1] Barua R, Munday RN. Intravascular angiomatosis in female urethral mass. Masson intravascular hemangioendothelioma. Urology 1983;21:191–3. 10.1016/0090-4295(83)90025-0 6681683

[2] Steffen C . The man behind the eponym: C. L. Pierre Masson. Am J Dermatopathol 2003;25:71–6. 10.1097/00000372-200302000-00015 12544105

[3] Tedla M, Bežová M, Biró C et al. Intravascular papillary endothelial hyperplasia of larynx: case report and literature review of all head and neck cases. Otolaryngologia polska = The Polish otolaryngology 2014;68:200–3. 10.1016/j.otpol.2014.03.002 24981303

[4] Carta F, Sionis S, Ledda V et al. Parotid Masson's tumor: case report. Braz J Otorhinolaryngol 2018;84:523–5. 10.1016/j.bjorl.2016.01.003 27150026 PMC9449239

[5] Hashimoto H, Daimaru Y, Enjoji M. Intravascular papillary endothelial hyperplasia. A clinicopathologic study of 91 cases. Am J Dermatopathol 1983;5:539–46. 10.1097/00000372-198312000-00004 6666836

[6] Abdal K, Hafezi Ahmadi M. Masson's hemangioma of the urethra: a case report. Iran J Med Sci 2018;43:336–9. 29892154 PMC5993900

[7] Essid MA, Bouzouita A, Blel A et al. Masson's tumor of the kidney: a case report. J Med Case Reports 2018;12:376. 10.1186/s13256-018-1898-2 PMC6303989 30577814

[8] Pauly M, Sruthi R, Subramanian K et al. Masson's tumor of the ocular surface - a rare clinical entity. Oman J Opthalmol 2022;15:370–2. 10.4103/ojo.ojo_162_21 PMC9905928 36760944

[9] Shi M, Zhao QH, Dai GG et al. Value of ultrasonography in diagnosis and classification of Masson's tumor. Zhonghua Yi Xue Za Zhi 2022;102:2295–7. 10.3760/cma.j.cn112137-20211229-02923 35927062

[10] Wagh VB, Kyprianou I, Burns J et al. Periorbital Masson's tumor: a case series. Ophthal Plast Reconstr Surg 2011;27:e55–7. 10.1097/IOP.0b013e3181e978f4 20829727

